# Characterization of host microRNAs that respond to DNA virus infection in a crustacean

**DOI:** 10.1186/1471-2164-13-159

**Published:** 2012-04-30

**Authors:** Tianzhi Huang, Dandan Xu, Xiaobo Zhang

**Affiliations:** 1 Key Laboratory of Conservation Biology for Endangered Wildlife of Ministry of Education, Key Laboratory of Animal Virology of Ministry of Agriculture and College of Life Sciences, Zhejiang University, Hangzhou, 310058, People’s Republic of China

**Keywords:** Invertebrate miRNAs, WSSV, Sequencing, EST assembly, Virus-host interaction, GO analysis, Evolution

## Abstract

**Background:**

MicroRNAs (miRNAs) are key posttranscriptional regulators of gene expression that are implicated in many processes of eukaryotic cells. It is known that the expression profiles of host miRNAs can be reshaped by viruses. However, a systematic investigation of marine invertebrate miRNAs that respond to virus infection has not yet been performed.

**Results:**

In this study, the shrimp *Marsupenaeus japonicus* was challenged by white spot syndrome virus (WSSV). Small RNA sequencing of WSSV-infected shrimp at different time post-infection (0, 6, 24 and 48 h) identified 63 host miRNAs, 48 of which were conserved in other animals, representing 43 distinct families. Of the identified host miRNAs, 31 were differentially expressed in response to virus infection, of which 25 were up-regulated and six down-regulated. The results were confirmed by northern blots. The TargetScan and miRanda algorithms showed that most target genes of the differentially expressed miRNAs were related to immune responses. Gene ontology analysis revealed that immune signaling pathways were mediated by these miRNAs. Evolutionary analysis showed that three of them, miR-1, miR-7 and miR-34, are highly conserved in shrimp, fruit fly and humans and function in the similar pathways.

**Conclusions:**

Our study provides the first large-scale characterization of marine invertebrate miRNAs that respond to virus infection. This will help to reveal the molecular events involved in virus-host interactions mediated by miRNAs and their evolution in animals.

## Background

MicroRNAs (miRNAs) are a large class of small non-coding RNAs that are found in diverse eukaryotic organisms. They range in size from 18 to 26 nucleotides and are cut sequentially from the stem regions of long hairpin transcripts by two RNase III proteins, Drosha and Dicer [1-3]. The mature miRNA strand is liberated from the miRNA:miRNA* duplex and incorporated into the RNA-induced silencing complex, where it controls the expression of cognate mRNA through degradation or translation repression [4-8]. It is known that miRNAs have important roles in many eukaryotic cellular pathways, including developmental timing, cell differentiation and proliferation, apoptosis, energy metabolism, cancer and immune defense [1,3,9-13]. Host miRNAs are believed to be key regulators of virus-host interactions [14-16]. To date, however, information about the pathways mediated by host miRNAs or their evolution is limited.

It has been reported that infections of some mammalian viruses can alter the host miRNA expression profiles, and the expression patterns of some host miRNAs change markedly over the time course of viral infection [15,16]. These changes reflect that the host miRNAs may have important roles in the virus-host interactions. These miRNAs may be involved in the host immunity to the virus invasion, or in virus infection to create favorable intracellular environments for virus replication. A systematic investigation of marine invertebrate miRNAs whose expression is altered in response to virus infection has not yet been performed [17]. Invertebrates, which do not possess a lymphocyte-based adaptive immune system, rely entirely on innate immunity.

Since the first miRNAs, lin-4 and let-7, were identified in *Caenorhabditis elegans* as potential regulators of animal development [18,19], 15,172 miRNAs have been discovered from various organisms (miRNA Registry, Release 17.0, April 2011), including mammals, plants, insects, nematodes and viruses. These miRNAs have been identified through computational or experimental approaches [20]. Many miRNAs are conserved among related species, suggesting that their functions may be evolutionarily conserved [1,21-23]. Using phylogenetic conservation and the criterion of a precursor hairpin structure (a characteristic hairpin structure with small internal loops, with the mature miRNA embedded in the stem of the hairpin), various computer programs have been developed to predict miRNAs, such as TargetScan [24], miRanda [25], MiRAlign [26] and Srnaloop [27]. However, the computational approaches are limited to organisms whose whole genome sequences are available. Recently, the high-throughput sequencing approach has successfully been used to identify miRNAs from various organisms [28-30]. Although this approach may omit the miRNAs with low abundance, it remains the approach of choice for identification of miRNAs in organisms whose whole genome sequences are unavailable [31]. Despite the large number of miRNAs that have been deposited in the miRBase database, this database is likely to be far from saturated as abundant miRNAs are still undiscovered from unexploited organisms. To date, identifications of miRNAs are limited to non-marine species, and very little information is available about the miRNAs of marine organisms.

In this study, the shrimp miRNAs involved in virus infection were investigated. Shrimps are one of the most important groups of species in marine aquaculture. In the past few decades, worldwide shrimp culture has been threatened by viral diseases, especially that by the white spot syndrome virus (WSSV) [32]. Owing to the lack of a true adaptive immune response system like that of vertebrates, invertebrates rely completely on the innate immune system to resist virus invasion. The miRNAs of invertebrates in general and marine invertebrates in particular, in response to virus infection, remain to be studied. In the present study, the miRNAs of WSSV-challenged shrimp (*M. japonicus*) were characterized. The results showed that 31 shrimp miRNAs defended against virus infection by regulating immune pathways. Some miRNAs were highly conserved in shrimp, fruit fly and humans and function in the similar pathways. Our study provides clues to the molecular events mediated by host miRNAs in host-virus interactions.

## Results

### Sequence analysis of shrimp miRNAs in response to WSSV infection

**Figure 1:**
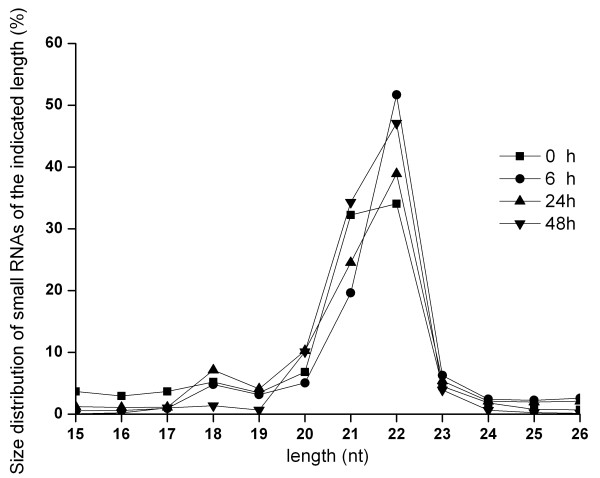
**Size distribution of small RNAs found by sequencing.** The symbols represented the time post- infection in hours.

To get an overview of the host miRNAs expressed in response to virus infection, the small RNAs of WSSV-infected shrimp at various times after infection were sequenced. After removal of mRNA, rRNA, tRNA, snRNA and snoRNA sequences, high-throughput sequencing generated a total of 35,588,792 raw sequences, of which 25,009,711 reads were mappable to miRBase 15.0 or to the shrimp GenBank expressed sequence tag (EST) database ( Additional file 1). However, no sequence mapped to the WSSV genome sequence. The analyses showed that the majority of the non-redundant sequences were 20–24 nucleotides (nt) in length, which is typical for products processed by the enzyme Dicer (Figure 1). Direct sequencing also revealed that the sequences at the 3′ ends of the miRNAs (52.7% of total reads) appeared to be more heterogeneous than those at the 5′ ends (18.9% of total reads), suggesting that the 5′ ends of miRNAs have key roles in target recognition, such as roles as seed sequences.

To characterize the shrimp miRNA homologs, the miRNA sequences were compared using BLAST with miRBase 15.0 with an E-value similarity cutoff of 1e–10. This revealed that 48 miRNAs [GenBank: JQ706251- JQ706298] were mapped to other known arthropod miRNAs and pre-miRNAs in miRBase 15.0. But the mapped pre-miRNAs could not be mapped to shrimp ESTs. Therefore, these sequences were identified as conserved miRNAs of shrimp and could be classified into 43 distinct families (Table 1 and Additional file 2). The remaining miRNAs with no homolog were compared using BLASTN with the shrimp EST database, allowing one or two mismatches between each pair of sequences. Hairpin structures were predicted in the mapped ESTs using the Mfold program. According to this criterion, a total of 15 miRNAs [GenBank: JQ706299-JQ706313] with no homolog in other animals were identified (Table 1 and Additional file 3), which might be specific to shrimp or not yet discovered in other animals. At different times after infection, different but overlapping sets of shrimp miRNAs were expressed (Table 1), indicating changes in host miRNA expression during virus infection.

**Table 1:** Shrimp miRNAs up-regulated or down-regulated in response to WSSV infection at different time post-infection

**Time post-infection**	**miRNAs conserved in animals**	**miRNAs with no homologue**
**0 h**	miR-1, miR-7, miR-9, miR-10a, miR-10*, miR-33, miR-34 miR-71, miR-79, miR-92a, miR-92b, miR-100, miR-133 miR-184, miR-252, miR-71*, miR-let7, miR-2a, miR-2b miR-2c, miR-8, miR-12, miR-87, miR-190, miR-193 miR-263a, miR-275, miR-276, miR-276b, miR-279, miR-281 miR-282, miR-305, miR-315, miR-317, miR-750, miR-965 miR-993, miR-1000, miR-276a*, miR-281-2*, miR-8*	miR-S1, miR-S2 miR-S3, miR-S5 miR-S6, miR-S10 miR-S12, miR-S15
**6 h**	miR-1, miR-7, miR-9, miR-10a, miR-10*, miR-33, miR-34 miR-71, miR-79, miR-92a, miR-92b, miR-100, miR-133 miR-184, miR-252, miR-71*, miR-let7, miR-2a, miR-2b miR-2c, miR-8, miR-12, miR-87, miR-190, miR-193 miR-263a, miR-275, miR-276, miR-276b, miR-279, miR-281 miR-282, miR-305, miR-315, miR-317, miR-750, miR-965 miR-993, miR-1000, miR-276a*, miR-281-2*, miR-8* miR-252b, miR-278, miR-981, miR-bantam, miR-2001	miR-S2
**24 h**	miR-1, miR-7, miR-9, miR-10a, miR-10*, miR-33, miR-34 miR-71, miR-79, miR-92a, miR-92b, miR-100, miR-133 miR-184, miR-252, miR-71*, miR-let7, miR-2a, miR-2b miR-2c, miR-8, miR-12, miR-87, miR-190, miR-193 miR-263a, miR-275, miR-276, miR-276b, miR-279, miR-281 miR-282, miR-305, miR-315, miR-317, miR-750, miR-965 miR-993, miR-1000, miR-276a*, miR-281-2*, miR-8* miR-252b, miR-13a, miR-981, miR-bantam, miR-2001	miR-S1, miR-S2 miR-S3, miR-S4 miR-S5, miR-S6 miR-S7, miR-S8 miR-S9, miR-S10 miR-S11, miR-S12 miR-S13, miR-S14 miR-S15
**48 h**	miR-1, miR-7, miR-9, miR-10a, miR-10*, miR-33, miR-34 miR-71, miR-79, miR-13a, miR-92b, miR-100, miR-133 miR-184, miR-252, miR-71*, miR-let7, miR-2a, miR-2b miR-2c, miR-8, miR-12, miR-87, miR-190, miR-193 miR-263a, miR-275, miR-276, miR-276b, miR-279, miR-281 miR-282, miR-305, miR-315, miR-317, miR-750, miR-965 miR-993, miR-1000, miR-276a*, miR-281-2*, miR-8* miR-252b, miR-278, miR-981, miR-bantam, miR-2001	miR-S1, miR-S2 miR-S3, miR-S4 miR-S5, miR-S6 miR-S7, miR-S8 miR-S9, miR-S11 miR-S12, miR-S13 miR-S14

### Host miRNAs involved in virus infection

**Figure 2:**
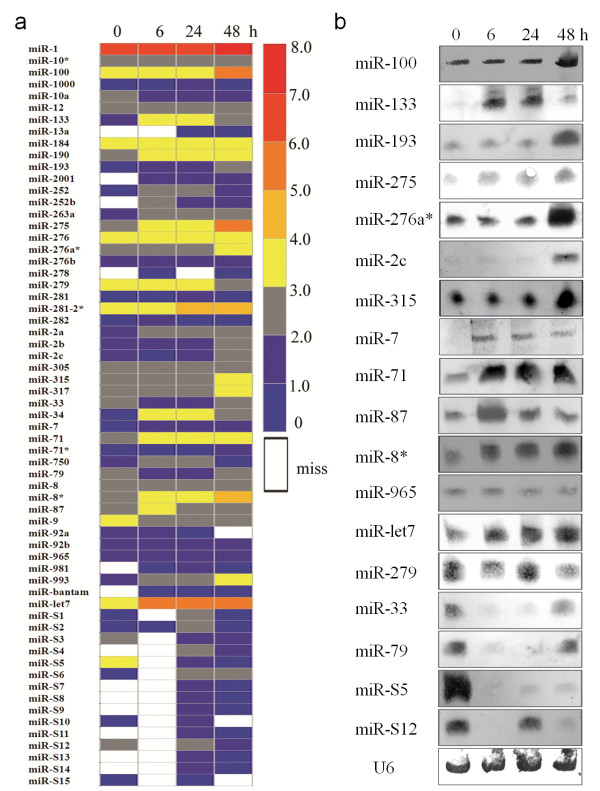
**Expression of shrimp miRNAs in response to viral infection.(a)** The miRNA expression profiles of WSSV-challenged shrimp at different time post-infection (0, 6, 24 and 48 h). The numbers on the right indicated the log10 of the number of copies of miRNAs. Miss, no copy detected. **(b)** Northern blots of selected shrimp miRNAs. Total RNA extracted from the lymphoid organs of the virus-free and WSSV-infected shrimp at different time post-infection (0, 6, 24 and 48 h) were blotted with DIG-labeled oligodeoxynucleotide probes. The probes were shown at the left. U6 was used as a loading control.

To characterize the host miRNAs involved in virus infection, the expression profiles of miRNAs of virus-free and WSSV-infected shrimp at various times after infection were compared. To assess the significance of the observed changes in miRNA counts between the two different libraries, the Audice-Claverie test, the Fisher exact test, and the Chi-squared 2 × 2 test were used, with a Bonferroni correction for multiple comparisons. A p-value < 0.01 indicated that differences in the miRNA counts were statistically significant. The results showed that the expression patterns of many miRNAs did not significantly change in response to the WSSV infection, but 31 miRNAs (total counts ≥200) were differentially expressed by more than twofold with a statistical significance of p < 0.01 (Figure 2a and Table 2). Comparison with the expression patterns of miRNAs at 0 h after infection showed that 25 miRNAs were significantly up-regulated by more than twofold: miR-1, miR-100, miR-133, miR-184, miR-190, miR-193, miR-252, miR-263a, miR-275, miR-276a*, miR-281-2*, miR-2a, miR-2b, miR-2c, miR-315, miR-317, miR-34, miR-7, miR-71, miR-8*, miR-87, miR-965, miR-993, miR-let7 and miR-S2. Six miRNAs (miR-279, miR-33, miR-79, miR-9, miR-S5 and miR-S12) were significantly down-regulated by more than twofold.

**Table 2:** Expression patterns of up-regulated or down-regulated shrimp miRNAs after WSSV infection

**MiRNA name**	**Counts of 0 hpi**	**Ratio of counts to 0 hpi at**		
		**6 hpi**	**24 hpi**	**48 hpi**
**up-regulation with ≥2 fold**		**up-regulation with ≥2 fold**			
miR-1	1292519	1.53	1.21	2.74	
miR-2a	97	2.85	2.42	3.62	
miR-2b	37	0.86	0.7	5.1	
miR-2c	15	0.56	0.73	23.46	
miR-7	6	15.16	16	14.16	
miR-34	9	174.55	120.11	18.77	
miR-71	590	2.85	2.19	9.03	
miR-87	229	5.23	2.32	2.96	
miR-100	5550	0.8	0.69	20.89	
miR-133	27	46.89	44.52	16.63	
miR-184	1082	8.16	6.4	4.51	
miR-190	389	3.67	2.79	6.69	
miR-193	3	24.6	13.3	42.6	
miR-252	7	36.29	34.14	8.14	
miR-263a	23	25.61	19.74	6.7	
miR-275	364	18.74	10.28	87.04	
miR-315	151	2.15	1.54	12.16	
miR-317	121	3.79	2.63	9.02	
miR-965	39	1.58	1.79	2.46	
miR-993	69	7.19	4.1	16.07	
miR-let7	6075	46.76	40.88	18.64	
miR-281-2*	1885	4.1	13.26	6.38	
miR-8*	787	6.42	3.82	13	
miR-276a*	459	1.46	1.09	14.15	
miR-S2	6	1.16	37	0.83	
**Down-regulation with ≥2 fold**			**Down-regulation with ≥2 fold**		
miR-33	631	0.04	0.03	1.29	
miR-79	895	0.04	0.03	0.19	
miR-9	6175	0.05	0.05	0.15	
miR-279	2752	0.71	0.85	0.24	
miR-S5	1063	0	0.06	0	
miR-S12	376	0	0.67	0.03	

Array data for 31 host miRNAs with high-quality data (total counts ≥200) at different time points after WSSV infection. hpi, hours post infection.

To confirm the involvement of these miRNAs in WSSV infection, 18 of them were selected at random for Northern blots. These showed expression patterns similar to those found by sequencing (Figure 2b); however, a little inconsistency was shown in miR-133, miR-193 and miR-2c compared with the results of sequencing, possibly owing to the low sensitivity of digoxigenin (DIG)-labeled oligodeoxynucleotide probes or for other unknown reasons.

### Pathways mediated by miRNAs

To facilitate the prediction of the miRNA target gene, shrimp ESTs (162,926 EST reads) were assembled. The results showed that the assembled ESTs could be used for the miRNA target gene prediction. To reveal the interactions between the host miRNAs and virus genes, a total of 232 3’ untranslated regions (UTRs) from the WSSV genome were used for target gene prediction. Analyses with the TargetScan and miRanda algorithms revealed the targets of 17 of the miRNAs that responded to virus infection (Table 3). The target genes were related to immune responses, gene expression regulation, signal transduction and metabolism. Some miRNAs, such as miR-34 and miR-S12, could target 7–8 genes. One of the predicted viral target genes of miR-7 was *wsv477*, an early gene that might have a key role in DNA replication and virus proliferation [33]. The miR-7 might act as a regulator of components of the immune system to inhibit virus replication through their direct interaction with viral mRNA. However, fewer viral genes than host genes were targeted by host miRNAs (Table 3), suggesting that the short typical length of viral 3’ UTRs evolved to minimize the effects of the host miRNAs.

**Table 3:** Target genes of miRNAs predicted by TargetScan and miRanda algorithms

**miRNA**	**overlapping genes**
**miR-1**	KRAB domain-containing zinc finger protein
**miR-12**	acyl-CoA binding domain containing 7
**miR-133**	ubiquitin-conjugating enzyme E2 A
**miR-13a**	O-methyltransferase; fibroinase; Mn superoxide dismutase; DNA-binding nuclear protein p8; peroxin-11 C
**miR-2001**	Scavenger receptor class B; glycoprotein 25 l; vp28 (WSSV)
**miR-263a**	Dihydropteridine reductase; Tetraspanins-like protein
**miR-275**	Peritrophin A; calcium and integrin binding protein CIB
**miR-276b**	Vacuolar ATP synthase
**miR-278**	eukaryotic translation initiation factor 3 subunit
**miR-279**	ubiquitin protein ligase; Exoskeletal protein
**miR-281**	eukaryotic translation elongation factor 1 alpha (eEF-1a); elongation factor EF-1 alpha subunit
**miR-282**	ribose 5-phosphate isomerase A; small subunit ribosomal protein S6e
**miR-2a**	Melanization interacting protein; Fortilin binding protein 1; O-methyltransferase
**miR-317**	hfb2 protein; (R)-3-amino-2-methylpropionate-pyruvate transaminase; Eukaryotic initiation factor 1A; maleylacetoacetate isomerase; hfb2 protein
**miR-34**	solute carrier family 37 (glycerol-3-phosphate transporter), member 2; Rpl6, NV12167; ribosomal protein L6; K02934 large subunit ribosomal protein L6e; ubiquitin protein ligase; K10573 ubiquitin-conjugating enzyme E2 A; Nuclear autoantigenic sperm protein; similar to elongase, putative; E3 ubiquitin ligase
**miR-7**	eukaryotic translation initiation factor 4A2; translation initiation factor eIF-4A; wsv477 (WSSV)
**miR-71**	actin 1; microsomal signal peptidase; signal peptidase complex subunit 3
miR-8*	hfb2 protein
**miR-87**	alanine-glyoxylate transaminase
**miR-92a**	Eukaryotic initiation factor 1A; maleylacetoacetate isomerase
**miR-let7**	rab11; T-complex protein 1 subunit gamma
**miR-S1**	Astacin
**miR-S10**	Rnps1 protein; RNA-binding protein with serine-rich domain 1; nuclear distribution protein NUDC; isopentenyl-diphosphate delta isomerase 1
**miR-S12**	ribosomal protein L5; Glucosyl/glucuronosyl transferase (Fragment); myosin heavy chain; Cationic trypsin-3 precursor Pretrypsinogen III; trypsin; eukaryotic translation initiation factor 2B; ribosomal protein L13A; microsomal glutathione S-transferase; glutathione S-transferase; methionyl aminopeptidase
**miR-S13**	similar to ribosomal protein L28; K02903 large subunit ribosomal protein L28e
**miR-S14**	putative beta-NAC-like protein; phosphatidylserine receptor
**miR-S3**	acireductone dioxygenase; ubiquitin C-terminal hydrolase; Duplex-specific nuclease
**miR-S5**	phosphatidylserine receptor; cystatin B
**miR-S6**	Glucosyl/glucuronosyl transferase
**miR-S9**	3-hydroxyisobutyryl-CoA hydrolase

**Figure 3:**
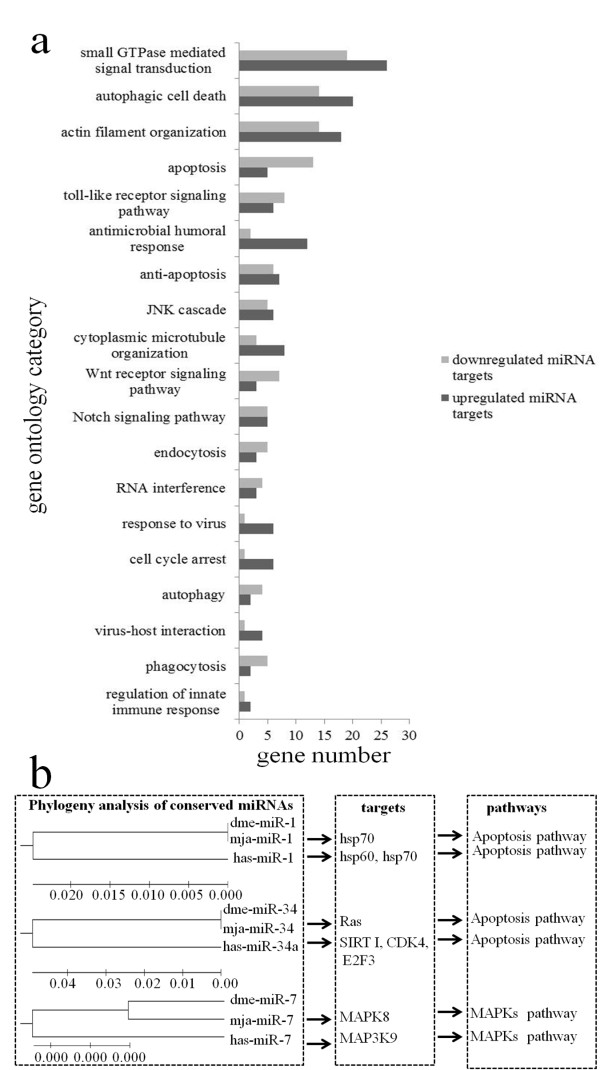
**Pathways mediated by miRNAs. (a)** GO analysis of genes targeted by miRNAs involved in virus-host interactions. Numbers indicated the total numbers of genes targeted by down- or up-regulated miRNAs in each putative functional category. **(b)** Pathways mediated by the miRNAs conserved with other animals. mja: *Marsupenaeus japonicus*; dme: *Drosophila melanogaster*; hsa: *Homo sapiens*.

To get an overview of the pathways mediated by host miRNAs, the target genes of miRNAs predicted by TargetScan were selected for gene ontology (GO) analysis. The results indicated that most of the target genes are involved in host immunity, including the small GTPase-mediated signaling transduction pathway, autophagy, phagocytosis, apoptosis, the Toll-like receptor signal pathway, antimicrobial humoral response, endocytosis, RNA interference (RNAi), response to viruses, virus-host interactions and regulation of innate immune response (Figure 3a). Moreover, a set of phagocytosis-related genes, such as myosin, actin, Arp2-Arp3 (Arp2/3), the serine/threonine kinase PAK and several members of the small G protein family, were enriched among the target genes of miR-79, suggesting that phagocytosis is an important immune strategy deployed by the host against virus infection [34]. Autophagy is an evolutionarily conserved mechanism of lysosomal degradation of unwanted cytoplasmic constituents, as well as of intracellular pathogens [35]. The GO analysis revealed that four genes targeted by miR-2 were classified into the autophagy subcategory, including the endogenous autophagosome marker protein microtubule-associated protein 1 light chain 3 (LC3), suggesting that miR-2 had important roles in the autophagy pathway.

The complementary binding between seed sequences of miRNAs and binding sites in target mRNAs might be conserved across species and might contribute to the functional conservation of miRNAs. Phylogenetic analysis, target gene prediction and pathway analysis showed that, among the 13 conserved miRNAs (miR-1, miR-100, miR-10a, miR-124, miR-125, miR-184, miR-33, miR-34, miR-7, miR-9, miR-92a, miR-92b and miR-let7), several highly conserved miRNAs (miR-1, miR-7 and miR-34) targeted the same or similar genes leading to the same pathways in shrimp, fruit fly and human (Figure 3b). This indicated that the beneficial miRNAs might be conserved during evolution because they aid survival.

## Discussion

As is well known, virus infection can disturb and subvert the host cellular processions and functions at several levels, such as changes in the expression of cellular transcripts, including miRNAs, and effects on the cell cycle or apoptosis of virus-infected cells [36]. During virus-host interactions, cellular miRNAs, which are key regulators of gene expression, are crucial [14]. However, we have not yet achieved a comprehensive view of the gene expression regulation mediated by miRNAs during virus-host interactions in marine invertebrates. In this study, an invertebrate shrimp was challenged by the DNA virus WSSV so as to characterize the host miRNAs involved in the response to virus infection. The results showed that 31 host miRNAs are involved in virus-host interactions, most of which are concerned with host immune responses. Our study provides the first large-scale characterization of marine invertebrate miRNAs and the pathways mediated by them in response to virus challenge.

Similar phenomena have been reported in mammals, the miRNA profiles of which are reshaped by hepatitis C virus (HCV), human immunodeficiency virus-1, human cytomegalovirus and Epstein–Barr virus [16,37-39]. The host miRNAs might be associated with the regulation of host immune systems or viral life cycles. RNAi knockdowns of Drosha and Dicer, two crucial proteins in animal miRNA biogenesis, resulted in a decrease in mature host miRNAs, which led to increased sensitivity of host to virus infection [15,40]. Some host miRNAs might thus represent antiviral miRNAs. When some putative antiviral miRNAs were blocked by locked nucleic acid-modified antisense oligoribonucleotides, the hosts failed to inhibit viral replication [11,41]. In some cases, host miRNA expression might be promoted by viruses to reshape the host intracellular environment to benefit viral replication [42]. In our study, phylogenetic analysis showed that the miR-1, miR-7 and miR-34 are highly conserved in shrimp, fruit fly and human and function in similar pathways. Our analyses predicted that miR-7, one of the miRNAs highly conserved between invertebrates and vertebrates, could target the mitogen-activated protein kinases (MAPKs), a situation identical to that in humans [43-45]. Recent studies revealed that MAPKs were activated by invading HCV, the orthopoxvirus vaccinia virus and visna virus, which aided viral replication [43-45]. Our analysis indicated that the WSSV early gene *wsv477* was also targeted by host miR-7, suggesting that host might inhibit virus infection by targeting viral transcripts with host miRNAs. It could thus be inferred that the functions of the conserved miRNAs have been preserved in animals during evolution. Because of the long evolutionary time since the divergence of shrimps and humans, studies on invertebrates would greatly benefit from even limited knowledge about shrimp virus-host interactions.

In our study, Solexa high-throughput deep sequencing was used to reveal the miRNAs involved in virus-host interactions. A total of 63 miRNAs were obtained, but no viral miRNA was revealed. This might be because of the small amounts of viral miRNAs. To characterize the viral miRNAs, an miRNA microarray could be used in the further studies.

## Conclusions

Our study provides the first large-scale characterization of marine invertebrate miRNAs in response to virus infection. The results showed that a total of 63 miRNAs of shrimp were obtained, 31 out of which were differentially expressed in response to virus infection. Among the differentially expressed miRNAs found, miR-1, miR-7 and miR-34 are highly conserved and mediate similar pathways, suggesting that some beneficial miRNAs have been preserved in animals during evolution. Invertebrates could therefore be good candidates for increasing our still limited knowledge about virus-host interactions because of their long evolutionary distance from vertebrates. Our study could help to reveal the molecular events of virus-host interactions mediated by miRNAs and their evolution in animals.

## Materials and methods

### Shrimp culture and WSSV infection

*M. japonicus* shrimp (10–15 g body weight) were cultured in groups of 20 individuals in each tank with artificial seawater and aeration. Before the experiments, the shrimp were maintained temporarily for 2–3 days and three shrimp were randomly selected for WSSV detection with WSSV-specific primers to ensure that the shrimp were virus-free. Then the virus-free shrimp were infected with WSSV at 10^4^ virions per ml by intramuscular injection using a syringe with a 29-gauge needle [46]. After WSSV challenge, the lymphoid organs of five individuals were collected at various times after infection (0, 6, 24 and 48 h) and immediately stored in liquid nitrogen for later use. Shrimp assays were conducted in accordance with COPE (the Committee on Publication Ethics).

### Sequencing of small RNAs

Total RNAs were isolated from the lymphoid organs of the virus-free and WSSV-infected shrimp at different times after infection using a mirVana miRNA Isolation Kit (Ambion, Austin, TX) according to the manufacturer’s instructions. The quantity and purity of total RNAs were monitored using a NanoDrop ND-1000 spectrophotometer (Nano Drop, DE) at a 260/280 ratio > 2.0. The integrity of total RNAs was analyzed using an Agilent 2100 Bioanalyzer system and an RNA 6000 Nano LabChip Kit (Agilent, CA) with an RNA integrity number (RIN) > 8.0. About 200 μg of total RNA was separated on a denaturing 15% polyacrylamide gel. The small RNAs (16–30 nt) were excised, quantified and precipitated with ethanol. After dephosphorylation by alkaline phosphatase, the purified small RNAs were ligated sequentially to RNA adapters (5′-ACAGGUUCAGAGUUCUACAGUCCGACGAUC-3′and 5′-UCGUAUGCCGUC UUCUGCUUG-3′). Reverse transcription and PCR amplification were performed after ligation. The resulting products were sequenced on the Genome Analyzer GA-I (Illumina, San Diego, CA) according to the manufacturer’s recommended protocol.

### Small RNA sequence analysis

Illumina's Genome Analyzer Pipeline software and the ACGT V3.1 program developed by LC Sciences (Houston, TX) were used for small RNA sequence analysis. The following sequences were removed: (1) sequences of the vector and adaptor, (2) low-quality sequences, (3) low-copy sequences (counts < 3), (4) sequences containing more than 80% A, C, G, or T, (5) sequences containing only A and C or only G and T, (6) sequences shorter than 16 nt and longer than 26 nt, (7) sequences containing 10 repeats of any dimer, 6 repeats of any trimer, or 5 repeats of any tetramer, (8) sequences matching mRNAs, rRNA, tRNA, snRNA, snoRNA. After these sequences were removed, all the remaining high-quality sequences were used for miRNA identification. To identify conserved miRNAs that were homologous with those of other species, all high-quality sequences were mapped to known mature and precursor arthropod miRNAs in miRBase 15.0 with an E-value similarity cutoff of 1e-10, and the pre-miRNAs were further mapped to the ESTs of the shrimp *Litopenaeus vannamei* from GenBank owing to the lack of the *Marsupenaeus japonicus* genome. To characterize novel miRNA candidates in shrimp, the remaining high-quality sequences with no homologs in miRBase 15.0 were analyzed by a BLASTN search against the shrimp EST database in the National Center for Biotechnology Information [47], allowing one or two mismatches between each pair of sequences. Hairpin RNA structures were predicted from the 65 nt sequences adjacent to the mapped ESTs in either direction by the MFOLD program using default parameters [48].

### Northern blotting

Total RNA was extracted from the lymphoid organs of the virus-free and WSSV-infected shrimp at different times post-infection (0, 6, 24 and 48 h) and quantified using a spectrophotometer (NanoDrop, Wilmington, USA). Then, 30 μg of total RNA was separated on a denaturing 15% polyacrylamide gel containing 8 M urea. The RNA was transferred to Hybond-N + membranes (Amersham Biosciences, Buckinghamshire, UK). After ultraviolet crosslinking (120 mJ, 30 s), the membrane was pre-hybridized in DIG Easy Hyb granules buffer (Roche, Basel, Switzerland) for 0.5 h, and this was followed by hybridization with a DIG-labeled DNA probe complementary to a specific miRNA sequence for 20 h. The DIG labeling and detection were performed following the manual of DIG High Prime DNA Labeling and Detection Starter Kit II (Roche).

### Shrimp EST assembly and 3' UTR extraction

Because of the lack of a shrimp genome sequence, the EST database containing 162,926 EST reads of the shrimp *Litopenaeus vannamei* from GenBank was used for the prediction of miRNA target genes [47]. However, most of the EST reads were too short (≤ 500 bp) to include information on 3' UTR sequences, which were the regions usually targeted by miRNAs. Therefore, the EST sequences were assembled using the CAP3 assembly program into a total of 31,831 non-redundant sequences comprising contigs and singlets [49,50]. According to the highest BLASTX and/or BLASTN hits, the most likely open reading frames were annotated, and their corresponding 3' UTRs were determined through to the polyadenylation signal. A poly(A) signal was taken as a sequence of ATTAAA or AATAAA located 10–35 nt from either the poly(A) tail or the end of the sequence. A poly(A) tail was taken as a run of at least six As at the end of a sequence. Incomplete 3' UTRs were removed from further analysis. To characterize the interactions between the host miRNAs and WSSV genes, a total of 232 3' UTRs from the WSSV genome sequence [GenBank: NC_003225] were extracted as described above.

### Prediction of genes targeted by miRNAs

To predict the genes targeted by miRNAs, two computational target prediction algorithms, TargetScan 5.1 and miRanda, were used [51,52]. The data-sets used were the assembled EST sequences and the 3' UTRs of WSSV. TargetScan was used to search for miRNA seed matches (nucleotides 2–8 from the 5' end of miRNA) in the 3' UTR sequences. miRanda was used to match the entire miRNA sequences. The miRanda parameters were set as free energy < −20 kcal/mol and score > 50. Finally, the results predicted by the two algorithms were combined and the overlaps were calculated.

### Gene ontology (GO) analysis

The coding sequences of the shrimp ESTs were extracted and used as queries to search the protein sequences collected by the GO database with the blast E value <1e-5 [53]. The best hit GO IDs were assigned to the shrimp EST sequences. The hypergeometric test statistic was then used to obtain the over-representation of particular functions or categories in the data of miRNA targets predicted by TargetScan 5.1 as compared with all the EST data. The P values were corrected by false discovery rate (FDR).

## Abbreviations

MiRNAs, MicroRNAs; WSSV, White spot syndrome virus; GO, Gene ontology; EST, Expressed sequence tag; NCBI, The National Center for Biotechnology Information; DIG, Digoxigenin; UTRs, Untranslated regions; FDR, False discovery rate; PAK, Serine/threonine p21-activated kinase; LC3, Microtubule-associated protein 1 light chain 3; HCV, Hepatitis C virus; HIV-1, Human immunodeficiency virus-1; HCMV, Human cytomegalovirus; EBV, Epstein-Barr virus; MAPKs, Mitogen-activated protein kinases; Mja, Marsupenaeus japonicus; Dme, Drosophila melanogaster; Hsa, Homo sapiens; RIN, RNA integrity number; Hpi, Hours post infection.

## Competing interests

The authors declare that they have no competing interests.

## Authors’ contributions

TZH performed the experiments, gathered and analyzed the data and drafted the manuscript. DDX performed some of the experiments and aided in writing the manuscript. XBZ designed the investigation, acquired and analyzed the data, and finished the final version of the manuscript. All authors carefully checked and approved this version of the manuscript.

## Supplementary Material

Additional file 1: Small RNA reads sequenced by Solexa technology from WSSV-infected shrimp at different time post-infection (0, 6, 24, and 48 h). Statistical analyses were based on the counts and percentages of the raw, mappable sequences and unique miRNA reads from virus-free WSSV-infected shrimps. Click here for file

Additional file 2: The shrimp miRNAs that were conserved in other animals. The 48 miRNAs conserved in other animals were classified into 43 distinct families. Click here for file

Additional file 3: The mapped EST sequences of shrimp-specific miRNAs. The mature sequence of each shrimp-specific miRNA was indicated as a different case in the fasta EST sequence. Click here for file
