# Supplementary material for: Characterization of host microRNAs that respond to DNA virus infection in a crustacean
Source: BMC Genomics. 2012 Apr 30;13:159. doi: 10.1186/1471-2164-13-159 (PMC3411463; doi:10.1186/1471-2164-13-159)
Supplement: Additional file 1: — Small RNA reads sequenced by Solexa technology from WSSV-infected shrimp at different time post-infection (0, 6, 24, and 48 h). Statistical analyses were based on the counts and percentages of the raw, mappable sequences and unique miRNA reads from virus-free WSSV-infected shrimps. [file 1471-2164-13-159-S1.doc]

|  | raw reads | mappable reads (%) | unique reads | mappable unique  reads (%) | unique miRNA  reads (%) |
| --- | --- | --- | --- | --- | --- |
| 0h | 8,660,116 | 5,431,241(62.72%) | 3,330,509 | 104,165(3.14%) | 2,444 (0.07%) |
| 6h | 8,204,617 | 5,205,219(63.44%) | 1,916,974 | 54,997(2.87%) | 1,564 (0.08%) |
| 24h | 8,772,020 | 5,551,405(63.29%) | 1,406,710 | 44,252(3.15%) | 2,370 (0.17%) |
| 48h | 9,952,039 | 8,821,846(82.61%) | 694,543 | 74,374(10.56%) | 2,070 (0.3%) |
| total | 35,588,792 | 25,009,711(82.61%) |  |  |  |
