# Supplementary material for: Characterization of host microRNAs that respond to DNA virus infection in a crustacean
Source: BMC Genomics. 2012 Apr 30;13:159. doi: 10.1186/1471-2164-13-159 (PMC3411463; doi:10.1186/1471-2164-13-159)
Supplement: Additional file 2: — The shrimp miRNAs that were conserved in other animals. The 48 miRNAs conserved in other animals were classified into 43 distinct families. [file 1471-2164-13-159-S2.doc]

| miRNA family | name | sequence (5'-3') | length (nt) |
| --- | --- | --- | --- |
| 1 | miR-1 | UGGAAUGUAAAGAAGUAUGGAG | 22 |
| 2 | miR-2a | UAUCACAGCCAGCUUUGAUGAGCG | 24 |
|  | miR-2b | UAUCACAGCCACCUUUGAUGAGCU | 24 |
|  | miR-2c | UAUCACAGCCAGCUUUGAUG | 20 |
| 7 | miR-7 | UGGAAGACUAGUGAUUUUGUUGUU | 24 |
| 8 | miR-8 | UAAUACUGUCAGGUAAAGAUGUA | 23 |
| 9 | miR-9 | UCUUUGGUGAUCUAGCUGUAUGA | 23 |
| 10 | miR-10a | UACCCUGUAGAUCCGAAUUUGU | 22 |
| 12 | miR-12 | UGAGUAUUACAUCAGGUACUGGU | 23 |
| 13 | miR-13a | UAUCACAGCCACCUUUGAUGAGCUU | 25 |
| 33 | miR-33 | AUGCAUUGUAGUUGCAUUGCA | 21 |
| 34 | miR-34 | UGGCAGUGUGGUUAGCUGGUUGU | 23 |
| 71 | miR-71 | UGAAAGACAUGGGUAGUGAGAU | 22 |
| 79 | miR-79 | AUAAAGCUAGAUUACCAAAGCA | 22 |
| 87 | miR-87 | GUGAGCAAAGUUUCAGGUGUGU | 22 |
| 92 | miR-92a | UAUUGCACUUGUCCCGGCCUGU | 22 |
|  | miR-92b | AAUUGCACUAGUCCCGGCCUG | 21 |
| 100 | miR-100 | AACCCGUAGAUCCGAACUUGUG | 22 |
| 133 | miR-133 | UUGGUCCCCUUCAACCAGCUGU | 22 |
| 184 | miR-184 | UGGACGGAGAACUGAUAAGGGC | 22 |
| 190 | miR-190 | AGAUAUGUUUGAUAUUCUUGGUUG | 24 |
| 193 | miR-193 | UACUGGCCUGCUAAGUCCCAA | 21 |
| 252 | miR-252 | CUAAGUACUAGUGCCGCAGGAG | 22 |
|  | miR-252b | CUAAGUAGUAGUGCCGCAGGUAA | 23 |
| 263 | miR-263a | AAUGGCACUGGAAGAAUUCACGG | 23 |
| 275 | miR-275 | UCAGGUACCUGAUGUAGCGCG | 21 |
| 276 | miR-276 | UAGGAACUUCAUACCGUGCUCU | 22 |
|  | miR-276b | UAGGAACUUUAUACCGUGCUCU | 22 |
| 278 | miR-278 | UCGGUGGGACUCUCGUCCGUUU | 22 |
| 279 | miR-279 | UGACUAGAUCCACACUCAUCCA | 22 |
| 281 | miR-281 | CUGUCAUGGAGUUGCUCUCUUU | 22 |
| 282 | miR-282 | UAGCCUCUCCUUGGCUUUGUCU | 22 |
| 305 | miR-305 | AUUGUACUUCAUCAGGUGCUCGG | 23 |
| 315 | miR-315 | UUUUGAUUGUUGCUCAGAAGG | 21 |
| 317 | miR-317 | UGAACACAGCUGGUGGUAUCUCAGU | 25 |
| 750 | miR-750 | CCAGAUCUAACUCUUCCAGCUCA | 23 |
| 965 | miR-965 | UAAGCGUAUGGCUUUUCCCCUC | 22 |
| 981 | miR-981 | UUCGUUGUCGUCGAAACCUGCAU | 23 |
| 993 | miR-993 | GAAGCUCGUUUCUACAGGUAUCU | 23 |
| 1000 | miR-1000 | AUAUUGUCCCGUCACAGCAGUA | 22 |
| 2001 | miR-2001 | UUGUGACCGUUAUAAUGGGCA | 21 |
| 10* | miR-10* | AAAUUCGGUUCUAGAGAGGUUU | 22 |
| 276a* | miR-276a* | AGCGAGGUAUAGAGUUCCUACG | 22 |
| 281-2* | miR-281-2* | AAGAGAGCUAUCCGUCGACAGU | 22 |
| 71* | miR-71* | AUCUCACUACCUUGUCUUUCA | 21 |
| 8* | miR-8* | CAUCUUACCGGACAGCAUUAGA | 22 |
| bantam | miR-bantam | UGAGAUCAUUGUGAAAGCUGAUUAU | 25 |
| let7 | miR-let7 | UGAGGUAGUAGGUUGUAUAGUU | 22 |
