# Supplementary material for: Characterization of host microRNAs that respond to DNA virus infection in a crustacean
Source: BMC Genomics. 2012 Apr 30;13:159. doi: 10.1186/1471-2164-13-159 (PMC3411463; doi:10.1186/1471-2164-13-159)
Supplement: Additional file 3: — The mapped EST sequences of shrimp-specific miRNAs. The mature sequence of each shrimp-specific miRNA was indicated as a different case in the fasta EST sequence. [file 1471-2164-13-159-S3.doc]

| name | sequence (5’-3’) | length (nt) |
| --- | --- | --- |
| miR-S1 | GGCACCGGACUGGCGCCCUU | 20 |
| miR-S2 | CGACGGAAAGGUGUCCAAGCUGG | 23 |
| miR-S3 | AGGUGUCCAGCCUCUGGUCGA | 21 |
| miR-S4 | CGAUAGAUCAAUGUAGGUAAGGGAA | 25 |
| miR-S5 | CGACCUCGAGUGGAGGGA | 18 |
| miR-S6 | AAUGGUCUAGUGAGGGCACCG | 21 |
| miR-S7 | GCGGUAGCCCGGGCAAGA | 18 |
| miR-S8 | GUUGACCGAAGCGGAGGAG | 19 |
| miR-S9 | ACGUCCGGCAGGUUUUACCCCU | 22 |
| miR-S10 | AGGACCUCGGUUCUAUUUUGUCG | 23 |
| miR-S11 | GGGGGCAUUCGUACUGCGACG | 21 |
| miR-S12 | AGGGGGAAACCGCGCUGAGCGUUA | 24 |
| miR-S13 | AGGGCGCGGUGGUGAGCGUA | 20 |
| miR-S14 | GGAGAGUUCAGCGAGCUGU | 19 |
| miR-S15 | AGUUGUGCAUGAAUGACU | 18 |
